# Research Priorities for a Circular Economy for Plastic Sustainability in the Caribbean

**DOI:** 10.1021/acs.est.5c16637

**Published:** 2026-10-19

**Authors:** Anne Marie Mozrall, Jessica P. Feliz Garrido, Pamela Tejada-Tejada, Akeem Mohammed, Brigitte Mildred Roldán Ramírez, Alana Malinde S.N. Lancaster, Iris Odett Atencio, Diego Batista, Denise Delvalle-Borrero, Christian Espinal, Ana De León, Sarra Gaspard, Ulises Jauregui-Haza, Phaedra C. Pezzullo, Mariely Ponciano, Jeffrey V. Smith, Laura Vásquez, Rafael Vasquez, Amy E. Landis

**Affiliations:** † 3557 Colorado School of Mines, Golden, Colorado 80401, United States; ‡ 3968 Michigan Technological University, Houghton, Michigan 49931, United States; § 37543 Instituto Tecnológico de Santo Domingo, Santo Domingo 10602, Dominican Republic; ∥ Department of Chemical Engineering, 37612 The University of the West Indies, St. Augustine Campus, St. Augustine 331131, Trinidad and Tobago; ⊥ Analytics and Research for Decision Making (ALIADO), 27983 University of Antioquia, Medellín 050010, Colombia; # The University of West Indies, Cave Hill Campus, Caribbean Environmental Law Unit, Bridgetown BB11000, Barbados; ¶ 252900 Technological University of Panama, Panama City 0819-07289, Panama; ∇ Laboratorio Nacional de Nanotecnología (LANOTEC), 672982 Centro Nacional de Alta Tecnología CENAT, San Jose 10109, Costa Rica; ○ Ministry of Industry, Commerce and MSMEs (MICM), Santo Domingo 10111, Dominican Republic; ⧫ Association of Industries of the Dominican Republic (AIRD), Santo Domingo 10109, Dominican Republic; †† Université des Antilles, Pointe-à-Pitre 97110, Guadeloupe; ‡‡ 1877 University of Colorado Boulder, Boulder Colorado 80309, United States; §§ Nueva Vida para los Residuos (NUVI), Santo Domingo 10109, Dominican Republic

**Keywords:** wider caribbean region, plastic pollution, waste management infrastructure, data scarcity, CEPlaSt network

## Abstract

Caribbean countries bear a disproportionate burden of marine plastic pollution because of their small landmass, high population density, and insufficient waste management infrastructure. Many Caribbean nations depend on informal, unmanaged landfills, underscoring an urgent need for more resilient, sustainable systems. This perspective synthesizes existing data to map the region’s plastic waste landscape and leverages regional expertise to identify key research priorities. This work emerged from interdisciplinary international professionals and members of the CEPlaSt (circular economy for plastic sustainability) network. A major barrier to circularity is the lack of reliable data on waste composition and flows, alongside stark infrastructure disparities across the wider Caribbean region. Addressing these gaps through targeted investment and region-specific planning is essential to modernize waste systems, reduce plastic emissions, and advance a sustainable Caribbean plastic circular economy.

## Graphical abstract

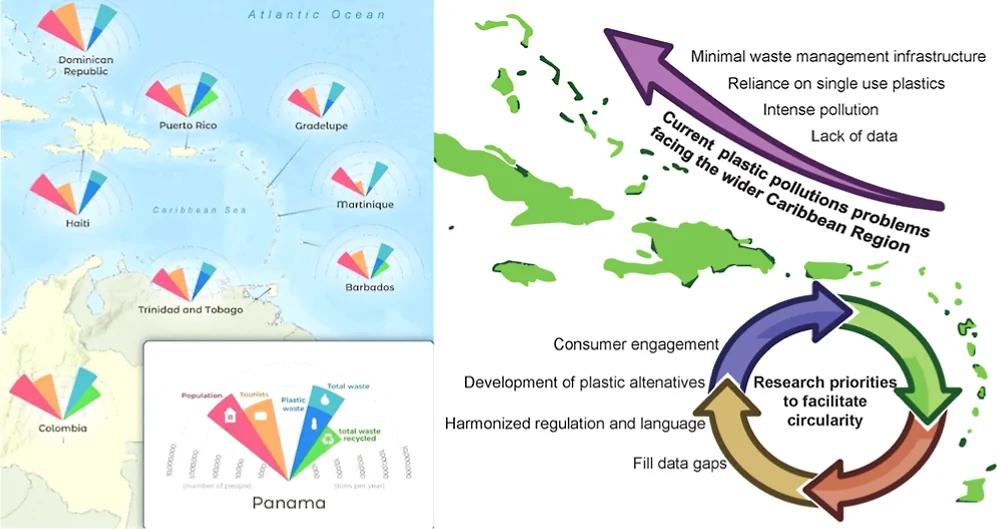

## Global and Caribbean Plastic Trends

Globally, only 9% of plastic waste is successfully recycled, while at least 22% is mismanaged and enters the environment as waste.^1^ In Latin America and the Caribbean, the proportion of plastic waste recycled is similar, with roughly 10% recovered. However, the Caribbean’s regional crisis is exacerbated by a mismanagement rate of 42%, which is nearly double the global average.^2,3^ Global plastic production currently amounts to 400 million tons per year and is set to double by 2040.^4^ However, Caribbean countries are primarily consumers and importers, with only about 4% of plastic consumed being produced within the region.^5^ It has been estimated that since 2010, between 4.8 and 12.7 million tons of plastic waste have been introduced into the marine environment globally,^1^ and more than 5 trillion pieces of plastic are estimated to be floating in the ocean.^6^ Although most plastic-related research focuses on plastic contamination of aquatic systems, current estimates suggest that plastic waste released to land was estimated to be 4–23× higher than that released to oceans.^7^ According to the United Nations (UN), 70% to 85% of the total debris in the Caribbean Sea comes from land-based activities, most of which comprises various plastic polymers.^8^ Consequently, the accumulation of plastic waste has become a global crisis, impacting biodiversity and the tourism industry^9,10^ and leading to the bioaccumulation of micro- and nanoplastics within regional food webs.^11,12^

Given the exceptional opportunities for improving plastic circularity and sustainability in the Caribbean, the circular economy for plastic sustainability (CEPlaSt) network, funded by the NSF, convenes experts from (number of participants from each discipline in brackets to follow) academia (26), government (4), and industry (5) to tackle the ongoing issue in the Caribbean that is plastic pollution. The authors of this perspective piece are CEPlaSt network members who convened at the 2024 Spring CEPlaSt Summit, held in Puerto Plata, Dominican Republic, to identify research priorities for the wider Caribbean region (WCR). Herein, WCR refers to any country that touches the Caribbean Sea. There were a total of 35 participants (number of participants from each country in brackets to follow), representing Barbados (1), Colombia (2), Costa Rica (1), the Dominican Republic (18), Guadeloupe (1), Jamaica (1), Panama (2), Trinidad and Tobago (2), and the United States (7). The CEPlaSt network aims to improve understanding of the plastic waste landscape and sustainable circular solutions in the WCR. In this perspective piece, we summarize the existing data and data gaps for plastic production and waste management and identify plastic research priorities for the region. Our strategy for identifying these priorities followed a systematic three-tier approach: first, establish a quantitative baseline of regional waste through a literature review (Figure 3); second, synthesize this data into an infrastructure profile (Table 1); and finally, use this evidence base to drive a structured expert elicitation to rank WCR research priorities (Figure 4). Despite the critical importance of transitioning toward a circular economy in the WCR, current literature remains largely fragmented, often focusing on localized waste management statistics rather than providing a cohesive, regional framework for systemic change. Comprehensive work that synthesizes these isolated data points into a unified strategic roadmap is lacking, leaving a significant gap in scholarly understanding of integrated plastic life-cycle solutions for the region. This study establishes a baseline by aggregating disparate data sets across the WCR. However, it also highlights a critical need to move beyond regional summation toward a comparative analysis of the unique geographical, economic, and logistical barriers, such as population density and remote waste accessibility, that define the plastic life cycle within these interconnected nations. By integrating quantitative material flow data with social and infrastructural research priorities, such as policy efficacy and waste management systems, this work explores the transdisciplinary framework necessary to bridge natural science and environmental engineering to advance holistic solutions for complex environmental systems.

**Table 1:** Percentage of Managed waste and waste Infrastructure in WCR^a^

Country	% of managed waste	Landfills	Curbside collection for trash	Material recovery and recycling facilities	Curbside collection for recycling	Waste to energy
Barbados	85%	one central, unlined informal landfill for the entire island	rural and urban collection	yes	collect on request, drop-in deposit, some industrial facilities	pilot sites
Colombia	81%	informal in both urban and rural areas formal in cities	urban collection	yes	urban Collection	yes, in cities
Costa Rica	89%	informal and formal in both urban and rural areas	rural and urban collection	yes, in cities	in larger cities and wealthy areas	n/d
Cuba	n/d	informal and formal in both urban and rural areas; state-of-the-art in Havana	rural and urban collection	yes, in cities	collection points in major cities	n/d
Dominican Republic	30%	informal landfills in both urban and rural areas	rural and urban collection	yes, in cities and private institutions	n/d	limited to private institutions
Guadeloupe	n/d	informal and formal in both urban and rural areas	rural and urban collection	n/d	n/d	n/d
Haiti	0%	informal in both urban and rural areas	n/d	n/d	n/d	n/d
Jamaica	n/d	informal and formal in both urban and rural areas	n/d	yes, in cities and mainly PET	n/d	n/d
Panama	n/d	informal and formal in both urban and rural areas	urban collection	yes	yes, just in cities; Ecospot	6% of total used tires incinerated in cement industry
Puerto Rico	n/d	informal and formal in both urban and rural areas	urban collection	yes	yes, about 20% of the population	proposed in Arecibo
Trinidad and Tobago	n/d	informal and formal in both rural areas	rural and urban collection	yes, in cities	n/d	n/d

a The authorship team reported the information herein, based on their review of the literature and the countries in which they reside and work.^37–42^ Table terminology: Informal landfills= unlined and unmanaged; Managed/formal landfills = lined and/or managed; state of the art = best available technology being used, as well as lined and managed; % of managed waste = percentage of waste collected within the country; waste to energy= energy recovery from waste, conversion of non-recyclable waste material into usable heat, electricity, or fuel.^43^ Ecospot = private recycling company such as Leafsinc^44^n/d = no data.

The amount of plastic found on WCR beaches far exceeds the global average, with 2014 items per kilometer of shoreline compared to 573 items per kilometer of shoreline in other countries.^13^ WCR countries are among the largest plastic polluters per capita globally.^14^ This is largely due to a lack of waste management infrastructure, reliance on single-use plastics for water and food, and an ever-growing tourism industry. Crucially, much of the mismanaged waste generated on these islands enters the ocean, only to wash back onto local shores, compounded by ocean currents that carry floating marine debris from external ocean sources into the region. WCR imports and consumption of plastics are disproportionate to their population, likely due to tourism.^15^ Within this context, plastic pollution reflects global patterns of colonialism.^16,17^ If you live in, or have visited the WCR outside of tourist areas, you may have seen plastic and other trash littering the shorelines (Figure 1A,D). However, you may also have seen someone cleaning the beaches to remove trash or other seaweed (Figure 1C). Alternatively, some beaches naturally do not suffer from ocean pollution (Figure 1B). The amount of debris on beaches around the WCR varies widely and thus poses different challenges from beach to beach.

**Figure 1:**
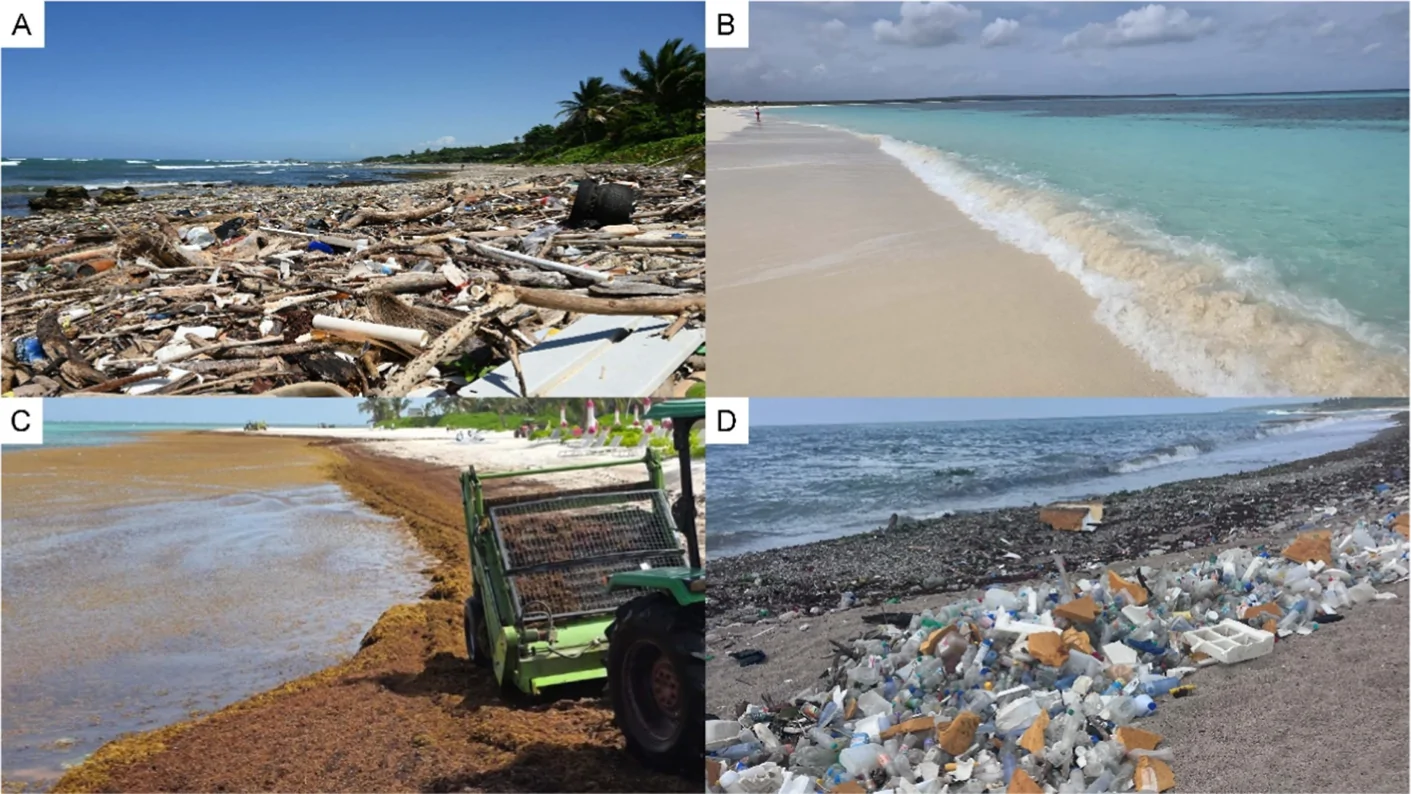
(A). Costa Dorada Beach in Puerto Plata, Dominican Republic. (B). Bahía de la Águilas, Dominican Republic. (C). Playa Blanca Punta Cana, Dominican Republic. (D). Playita de Nigua, Dominican Republic. The images above show the variability in plastic pollution on beaches around the Dominican Republic. Picture A credit: Aracelis Mena, taken at the 2024 CEPLaSt Summit.

While WCR countries often struggle with similar issues, the waste management landscape remains highly heterogeneous across the various islands and mainland nations. As a region, waste management in the WCR is far less studied than in other island regions worldwide. Many WCR nations are characterized by their prevalence of vertederos, which are unlined and unmanaged informal dumpsites, often situated dangerously close to fluvial corridors (Figure 2).^18–22^ These informal waste sites pose a significant environmental risk, as their lack of containment infrastructure allows contaminants to leach directly into the soil and debris to flow unhindered into major river systems. The region does not follow a single waste profile. Instead, it is a complex mosaic: some nations rely on these informal systems, while others use more modern waste management strategies. Even with formal infrastructure, many smaller island nations struggle with a critical lack of capacity, where a single primary landfill is often overwhelmed by the sheer volume of modern waste generation, further complicating the transition toward a circular and sustainable management framework.

**Figure 2:**
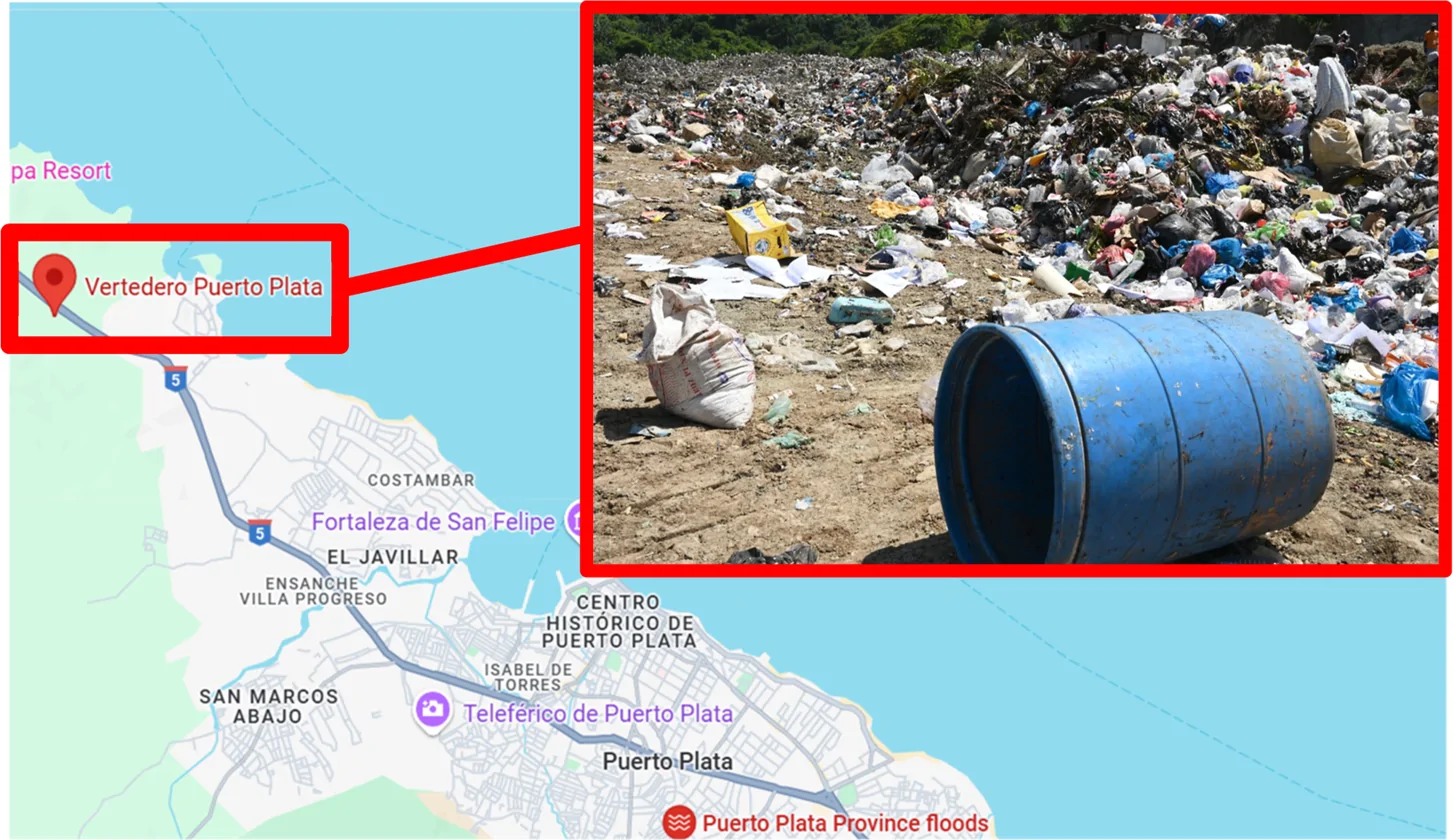
Informal landfill/open dumpsite or vertedero outside of Puerto Plata, Dominican Republic. Puerto Plata has a population of 321,597.^23^ Picture credit: Aracelis Mena, taken at the 2024 CEPlaSt Summit.

Most countries in the WCR are participating in ongoing negotiations for the Global Plastic Treaty, an international legally binding instrument emerging from the 2022 UN Environment Assembly resolution to address plastic pollution through a circular economy approach. The treaty aims to tackle issues such as phasing out single-use plastics, mitigating microplastics pollution, improving product design and waste management infrastructure, and ensuring a just transition for developing nations. Although negotiations remain unresolved, with major debate over plastic production limits, financing, and implementation, the Global Plastic Treaty could transform global waste management.

### Plastic Waste in the Caribbean

One of the primary barriers to plastic sustainability in WCR is the systemic lack of modern waste management infrastructure. This physical gap directly correlates with poor data-tracking systems, creating a cycle in which missing infrastructure prevents accurate reporting and a lack of data hinders the development of new facilities. Consequently, current waste profiles are characterized by significant data gaps, aging statistics, and inconsistent reporting methodologies and language across the region. Figure 3 represents the most recent available plastic and waste information at the time of writing. For most WCR countries, available information is limited to basic municipal solid waste (MSW) generation. Detailed metrics, specifically plastic waste as a fraction of total waste and recycling profiles, are inconsistent and at times scarce, with only 50% of the countries reporting total waste recycled. Furthermore, data on recycled plastic waste, plastic imports, and plastic exports were scarce for all nations, and therefore they were not depicted in the figure. In addition to data gaps, much of the data is considerably dated. Several data points are missing, mainly for total waste and plastic waste recycled in each country. These missing or partial data reports are not merely administrative oversights; they directly reflect the resource constraints and infrastructure deficits currently facing the WCR. For a detailed breakdown of data sources, years, and data-quality assessments, see the Supporting Information.

**Figure 3:**
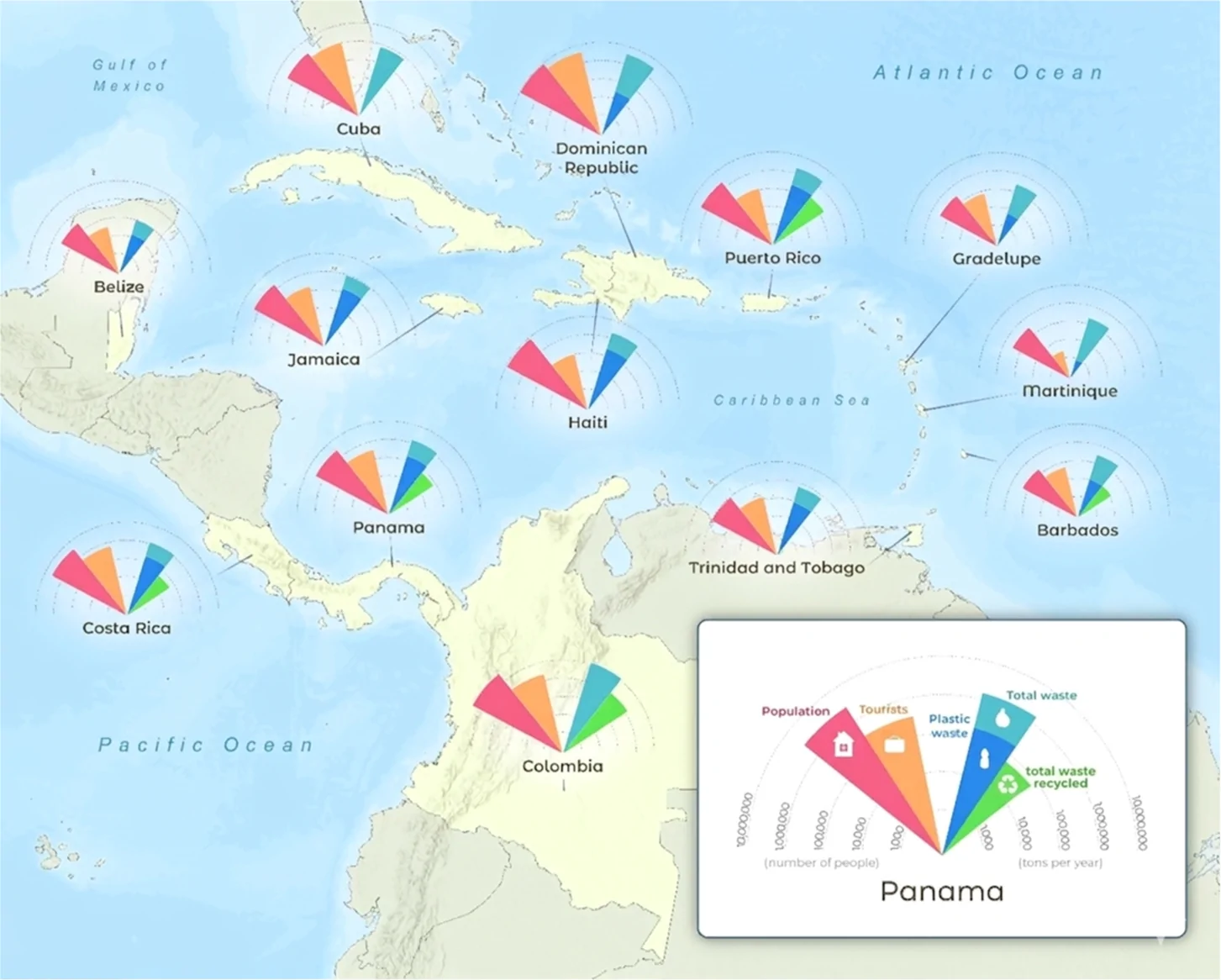
Waste profiles by country in comparison to population (mil. of people) and tourism (mil. of people per year). The key shows Panama as an example; the arcs have the same units for population and tons for all figures on the map. This figure highlights gaps in available data; total waste recycled (green) was not available for half of the countries. The data in Figure 3 represents the most recent available data at the time of writing. The data cover 2015–2024, with a few outliers: Trinidad and Tobago’s “Plastic waste per year” from 2009 and Puerto Rico’s “Total Waste” and “Plastic Waste” from 2003. The data presented in Figure 3 was aggregated from the World Tourism Organization, the World Bank Group, the United Nations Development Programme, and the Solid Waste HUB and Circular Economy.^24–27^

Major data gaps remain in quantifying and classifying waste production and management for nearly all WCR countries. Countries in WCR collect and manage waste data using diverse methodologies, making comparisons difficult. Even in countries with progressive waste management frameworks, administrative success does not always translate to comprehensive data availability. For instance, since 2010, Costa Rica has created a robust system that currently collects and manages 84% of anthropogenic waste through formalized collection processes.^24^ The collection framework places responsibility for waste management under the ministry and connects the whole waste management system.^24^ The system has created an organized inventory of residues and has made waste management decision-making more transparent and accessible to the public.^28–30^ However, while the Costa Rican system excels at tracking general waste streams, it still has gaps in quantifying specific waste streams. Similarly, in Colombia, efforts are underway to organize key waste handlers, such as recyclers, collection centers, and solid waste disposal/treatment facilities, to ensure information becomes official, standardized, and uniform across the sector. National public policies and regulations support these efforts. For example, the National Policy for Comprehensive olid Waste Management with a circular economy approach aims to improve waste management to minimize environmental impact, reduce landfill disposal, encourage waste separation at the source, and coordinate stakeholders to extend product life cycles.^31^ Other initiatives in Colombia include the single-use plastics law, extended producer responsibility (EPR), and regulations on packaging and container management. These measures help regulate and strengthen the development of the necessary infrastructure for effective waste management.^32^ Yet despite these policy foundations, standardized reporting is still in its infancy; official data on total plastic waste tonnage remains incomplete. These examples show a regional trend: even when “high-quality” policy and collection infrastructure exist, granular reporting to track specific materials like plastic is often lacking.

Standardized collection methodology and unified terminology are not merely academic goals and will significantly improve data comparability in the region. ***Better data comparability improves operational efficiency, creates regional economies of scale, and identifies opportunities for targeted intervention***. Countries specifically need granular, localized data to design and plan waste management infrastructure adequately. Census surveys, national polling, and artificial intelligence could improve data collection on tourism, plastic imports and exports, waste production, waste disposal, and the availability and access to different waste management systems.^33,34^ Currently, global waste management ranking systems, such as the Environmental Performance Index (EPI) and the Organization for Economic o-operation and Development (OECD), provide a high-level assessment of waste management globally and in several Caribbean countries. However, they do not account for the physical availability of waste management infrastructure, a major obstacle for the WRC region. To bridge this gap, the region must prioritize accurate, consistent collection and reporting of data such as plastic types, import/export volumes, and leakage rates. Further standardization of these metrics will serve goals beyond comparison, such as creating economies of scale, calibrating infrastructure, and targeted pollution intervention. Ultimately, without a current and extensive baseline of waste data across the region, it will be difficult to measure intervention success.

### Waste Management Infrastructure in the Caribbean

Available and accessible waste management infrastructure in the Caribbean varies widely, as summarized in Table 1, which presents the consensus on the current state of waste management in WCR countries based on CEPlaST Summit participants’ knowledge and existing literature. To ensure a comprehensive regional perspective, we supplemented and cross-referenced the qualitative findings from CEPlaST Summit participants with the literature. Some countries (Barbados, Colombia and Costa Rica) have managed or formal infrastructure throughout. In contrast, at the other end of the spectrum, some countries have mostly informal landfills with no coordinated collection and minimal recycling.

Although it may seem obvious, infrastructure is pivotal to effective waste collection, treatment, and management. No amount of consumer education, research, or cleanup efforts can replace infrastructure. However, Caribbean countries lack infrastructure for several reasons. First, waste infrastructure and management often correlate with economic development, suggesting that increased economic development in the WCR would lead to more investment in waste management infrastructure. Another challenge that the WCR faces is land availability for said infrastructure. Many WCR countries are islands and have relatively small land areas, which makes it difficult to find space for new facilities.

Many WCR countries require significant capital investment and logistical planning to modernize their waste management infrastructure; however, modernization does not necessarily mean adopting a one-size-fits-all approach, as is common in high-income or industrial nations. While curbside recycling collection is a standard metric for success in the Global North, it may not be the most efficient or culturally appropriate model for the WCR’s diverse geographic and economic landscapes. Currently, a significant waste-collection disconnect exists; even in WCR countries with material recovery facilities, almost none have organized curbside infrastructure. Rather than forcing a curbside model, future research should explore monetary incentives and alternative collection strategies, such as decentralized, community-led, or informal -sector integrated systems.^35,36^ Countries with more available and formalized infrastructure tend to report a higher percentage of managed waste. Yet, even for the exceptional performers, Table 1 reveals that all WCR countries have some informal landfills. Finally, very few landfills in WCR countries use waste-to-energy. Existing programs primarily operate at pilot scale or exist only as proposed plans, highlighting a gap between high-level policy ambition and the practical, localized infrastructure needed to handle the region’s specific waste profile.

### Looking Forward: Research Priorities for the Wider Caribbean Region

CEPlaST Summit expert participants identified plastic waste management needs and research topics that are most important to WCR through a facilitated expert elicitation process adapted from RCSA’s Scialog.^45^ These priorities were then condensed and prioritized by summit participants; the results are shown in Figure 4.

**Figure 4:**
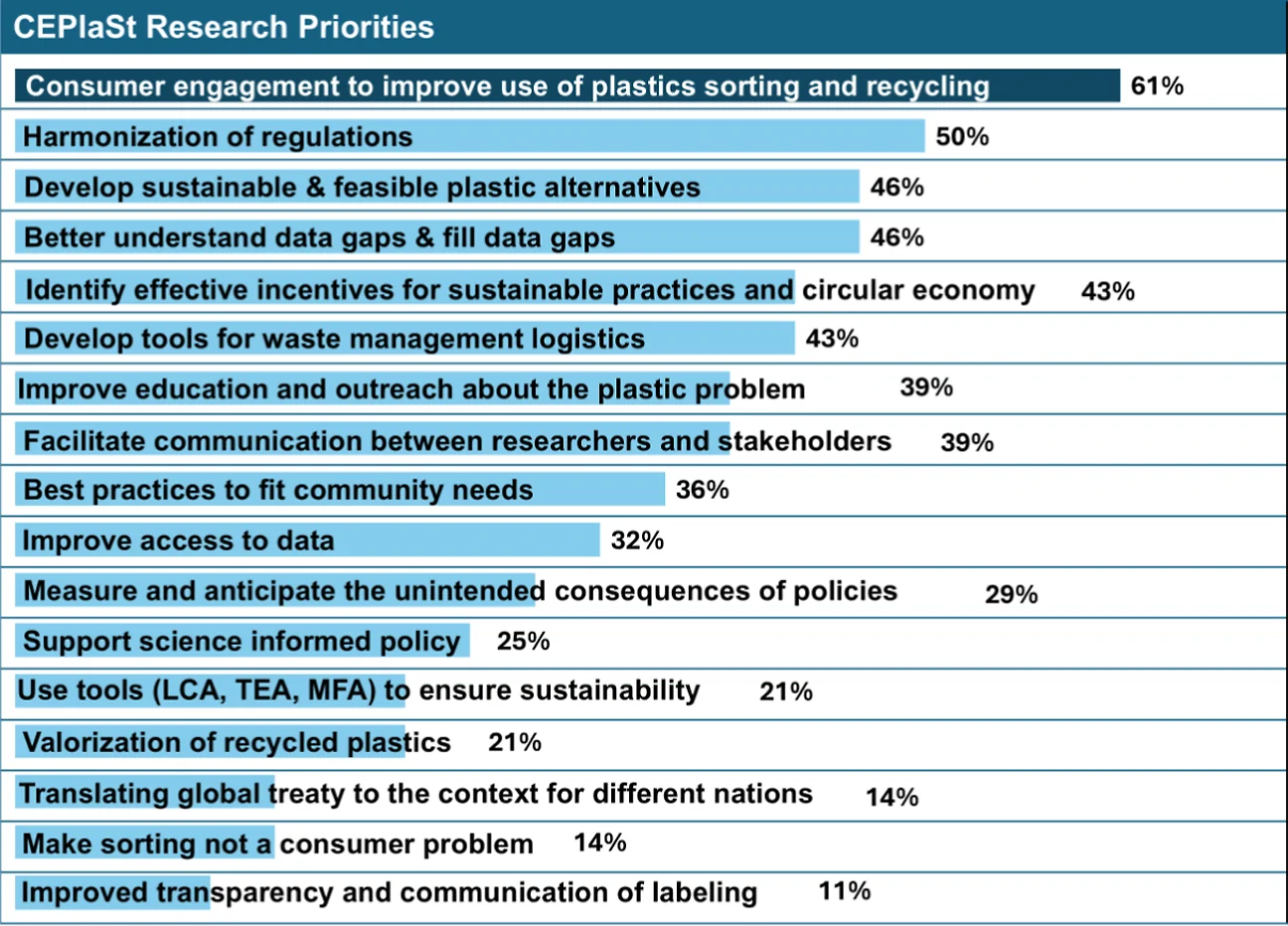
Research priorities identified and voted on by Summit participants. Research priorities were identified via an expert elicitation process developed by the Research Corporation for Science Advancement (RCSA) for their Scialog program.^45^ Percentages indicate the number of experts who agreed that this was a top priority for the Caribbean. Each of the 35 regional expert participants received five votes to distribute among the 17 research priorities the summit participants identified. Participants could put all their votes on one research priority or split them. Note that the top priority, consumer engagement, is not meant to shift sole responsibility onto end-users; rather, this priority calls for public education in the WCR as a tool for political mobilization, empowering citizens to demand accountability and improved waste management infrastructure for recycling.

Summit participants ranked consumer education as the highest priority. However, this priority, as written, comes with much discussion and caveats. Summit participants and authors recognize that no amount of consumer education on what to recycle or how to recycle will fix the WCR’s lack of waste management and recycling infrastructure. Rather than shifting responsibility onto end-users, public education in the WCR must be leveraged as a tool for political mobilization, empowering citizens to demand producer accountability, institutional transparency, the legislative adoption of an extended producer responsibility (EPR) framework, and improved waste management infrastructure for sorting and recycling. It has been documented in the literature that robust public education campaigns often lead to critical citizen-driven policy feedback loops, effectively forcing environmental issues onto national legislative agendas.^46,47^ However, the efficacy of public pressure as a driver for infrastructure investment is contingent on the varied socio-economic stability and political responsiveness of individual WCR nations, where competing priorities and resource constraints often hinder the implementation of environmental policies.^34^

The second priority focused on harmonization of regulations across the WCR.^34^ In this context, harmonization does not necessarily mean all countries need the same regulation. Rather, the goal is to develop a regional framework of best practices and model policies that individual countries can draw on, based on their specific needs. This is particularly critical for nations with minimal existing waste legislation, as it provides a baseline of effective strategies tailored to the unique constraints of island nations and small economies. Additionally, participants emphasized that while regulatory language should remain flexible, data collection methodologies for plastics and waste must align. Beyond domestic policy agreement, regional regulatory harmonization serves as a vital trade control mechanism. For import-dependent island nations, unified product standards and restricted material-entry lists provide the collective leverage needed to prevent the dumping of nonrecyclable or problematic plastic formats into the WCR.

One goal of the CEPlaSt Summit was to discuss how the Global Plastic Treaty would impact the Caribbean. Caribbean experts at the CEPlaSt Summit indicated that the Global Plastic Treaty should focus on supporting harmonization of regulations, facilitating the sharing of best practices (both policy and technical), and supporting or incentivizing sustainable infrastructure, such as material recovery facilities and plastic recycling and upcycling. Some of the research priorities for the WCR identified in Figure 4 are, at the time of writing, reflected in the draft of the global plastic treaty. Specifically, the treaty aligns with regional priorities around harmonizing regulations, developing sustainable and feasible plastic alternatives, addressing data gaps to support science-informed policy, and translating global treaty mandates into the localized context of different nations.^48–50^

A key issue CEPlaSt experts address is that “plastics” covers a wide range of materials, from the macro to the nano scale, and ranges from plastic packaging to plastics used in electronics and construction. The third-ranked research priority, “Develop sustainable and feasible plastic alternatives”, does not mean developing more complex plastics. The increasing complexity of new plastic materials has hindered sorting or recycling plastic waste. Simplification and standardizing plastic product design is an essential prerequisite for efficient waste management.^51^ Designing plastics that work in mechanical recycling systems can be a cost-effective way to recover and recycle plastics. To ensure long-term viability, the region must pair economies of scale with region-wide material standardization and eliminate nonrecyclable multimaterial formats.^34^ In addition, WCR countries should consider restrictions on material imports aligned with existing local waste management infrastructure. Furthermore, researchers must develop and validate sustainable alternatives tailored to the region’s unique resources. While global research drives polymer science, it should be coupled with feasibility assessments to ensure alternatives fit local waste challenges. An example is the successful jute bag industry in Bangladesh, which helps reduce single-use plastic bags.^52,53^ Another example is producing bioplastics from *Sargassum* seaweed, which can then be used to manufacture products like agricultural films, human and animal food packaging, feed/grain bags, dry food pouches, snack wrappers, and dairy or cheese wrappers.^54,55^ Sargassum-based products are currently in various stages of technological readiness. However, with the abundance of the seaweed in the Caribbean, researchers are looking to turn the current nuisance into an asset.^34^ While sargassum-based bioplastics represent a compelling circular opportunity to convert an ecological crisis into an economic asset, scaling requires overcoming critical roadblocks, including seasonal biomass variability^56^ and the current absence of industrial composting infrastructure across the region.^57^

In capital-constrained economies, robust data infrastructure is a fundamental prerequisite for mitigating risk and allocating capital. High-resolution waste audits and mandatory corporate disclosures are critical not only for optimizing municipal resource distribution, but also for unlocking climate finance and international development capital demanding measurable baselines. Governments need rigorous data to ensure they tackle the biggest plastic problems with solutions that deliver the highest return on investment. The WCR would benefit from cohesive plastic data collection and analysis procedures, including waste audits, improved reporting requirements for companies related to plastic production and consumption, and improved reporting and assessment of municipalitie’s waste generation and handling. Moving forward, the CEPlaSt network will deepen its regional impact by fostering cross-border collaboration with established organizations such as the Asociación Dominicana de la Industria de Plásticos (ADIPLAST),^58^ the Caribbean Biodiversity Fund,^59^ and Caribe Circular^60^ to synchronize policy responses and leverage shared development funding against our common plastic challenges.

Plastic waste disproportionately impacts Caribbean countries, largely driven by the exponential increase in plastic production and the WCR’s lack of modern waste management infrastructure. Existing global solutions often fail to account for the specific factors that define the WCR: namely, high dependence on plastic-packaged imports, limited space, and waste volume increases due to tourism. A significant lack of data on plastic and waste remains prohibitive to future planning for circular economies and sustainable development.

Authors from the WCR note that consumer engagement to improve policy and infrastructure for plastic sorting and recycling, harmonization of regulation, development of sustainable and feasible plastic alternatives, identification and filling of data gaps, and identification of effective incentives for sustainable practices and circular economy are major priorities for research and policy to advance circularity and sustainability for plastics. The research priorities presented herein highlight the needs of small island developing nations and should be considered by both local policymakers and global instruments such as the global plastic treaty. Future research should transition from quantifying data gaps to investigating how shared regional challenges and unique socioeconomic drivers can be leveraged to develop scalable, collective solutions that transcend the sum of individual national efforts. Finally, while the global plastic treaty has great potential to drive global efforts to reduce plastic pollution, a plan tailored to the WCR should prevent the burden of plastic waste from falling on countries with limited waste management capacity and promote regional and international knowledge exchange to build the foundation for an effective transition.

## Supplementary Material



## Notes

This material includes a raw data table, a simplified data table, and data collection methodology for Figure 3, along with a data quality ranking system and associated references (PDF)
