# Supplementary material for: Research Priorities for a Circular Economy for Plastic Sustainability in the Caribbean
Source: Environ Sci Technol. 2026 Oct 19;60(43):30345–54. doi: 10.1021/acs.est.5c16637 (PMC13738801; doi:10.1021/acs.est.5c16637)
Supplement: Supplementary file 1 [file es5c16637_si_001.pdf]

Title: *Research Priorities for a Circular Economy for Plastic Sustainability in the Caribbean*

Authors: Anne Marie Mozrall[1], Jessica P. Feliz Garrido[2], Pamela Tejada-Tejada[3], Akeem Mohammed[4], Brigitte Mildred Roldán Ramírez[5], Alana Malinde S.N. Lancaster[6], Iris Odett Atencio[7], Diego Batista[8], Denise Delvalle-Borrero[7], Christian Espinal[9], Ana De León[10], Sarra Gaspard[11], Ulises Jauregui-Haza[3], Phaedra C. Pezzullo[12], Mariely Ponciano[13], Jeffrey V. Smith[4], Laura Vásquez[3], Rafael Vasquez[9], Amy E. Landis[2]

[1] *Colorado School of Mines, Golden, Colorado 80401, United States*
[2] *Michigan Technological University, Houghton, Michigan 49931, United States*
[3] *Instituto Tecnológico de Santo Domingo, Santo Domingo 10602, Dominican Republic*
[4] *The University of the West Indies, St. Augustine Campus, Department of Chemical Engineering, St. Augustine, Trinidad and Tobago*
[5] *University of Antioquia, Analytics and Research for Decision Making (ALIADO), Medellín 050010, Colombia*
[6] *The University of the West Indies, Cave Hill Campus, Caribbean Environmental Law Unit, Bridgetown BB11000, Barbados*
[7] *Technological University of Panama, Panama City 0819-07289, Panama*
[8] *Laboratorio Nacional de Nanotecnología (LANOTEC), Centro Nacional de Alta Tecnología CENAT San José 10109,* Costa Rica
[9] *Ministry of Industry, Commerce and MSMEs (MICM), Santo Domingo 10111, Dominican Republic*
[10] *Association of Industries of the Dominican Republic (AIRD), Santo Domingo 10109, Dominican Republic*
[11] *Université des Antilles, Pointe-à-Pitre 97110, Guadeloupe*
[12] *University of Colorado Boulder, Boulder 80309, Colorado, United States*
[13] *Nueva Vida para los Residuos (NUVI), Santo Domingo 10109, Dominican Republic*

Data Quality Ranking System

1. ***High Quality (HQ-Green)***: stands out as an exception, providing comprehensive and recent waste composition audits with high transparency
2. ***Moderate Quality (MQ-Yellow)***: Provides consistent reporting with no missing data points, but some data is dated.
3. ***Poor Quality (PQ-Orange)***: Data is considerably dated with limited, missing, or undescriptive data
4. ***Incomplete/Nonexistent (I/N-Red)***: Several major data points missing

Raw Data for Figure 3

Table 1: *Raw data for Figure 3, showing the exact number for each data point, the source, and the year from which the data is from. Plastic recycled (tons/year), plastic produced (tons/year), and plastic consumption (tons/year) were considered but could not be found for any of the countries. Population is presented in millions of people (mil.), tourists in millions of people per year (mil. per year), and all values are rounded to the nearest thousand. Blacked-out spaces indicate data values that were not available.*

Country	***Ranking System***	***Total Waste (tons per year)***	***Source***	***Plastic waste (tons per year)***	***Source***	***Total waste recycled (tons per year)***	***Source***	***Pop. (mil.)***	***Source***	***Tourist (mil. per year)***	***Source***
Dominican Republic		4,290,000	(Montero et al. 2023)	26,938	(Centro de Capacitacion			11.2	(World Bank Group 2023)	7.163	(The World Tourism

	PQ		**DATA DATE 2021**		e Investigacion del Plastico 2022) **DATA DATE 2021**				**DATA DATE 2023**		Organization 2022) **DATA DATE 2022**
Colombia	PQ	14,220,000	(Montero et al. 2023) **DATA DATE 2021**			1,872,774	(Solid Waste HUB and Circular Economy 2021) **DATA DATE 2021**	52.8	(World Bank Group 2023) **DATA DATE 2023**	4.078	(Colombian Ministry of Commerce 2024) **DATA DATE 2023**
Panama	HQ	1,971,000	(United Nations Development Programme (UNDP) 2023) **DATA DATE 2021**	204,984	(United Nations Development Programme (UNDP) 2023) **DATA DATE 2021**	98,550	(Netherlands Enterprise Agency: Ministry of Foreign Affairs 2021) **DATA DATE 2017**	4.5	(World Bank Group 2023) **DATA DATE 2023**	0.549	(The World Tourism Organization 2022) **DATA DATE 2022**
Costa Rica	PQ	1,620,000	(Montero et al. 2023) **DATA DATE 2021**	202,500	(Montero et al. 2023) **DATA DATE 2021**	63,990	(Solid Waste HUB and Circular Economy 2021) **DATA DATE 2021**	5.3	(World Bank Group 2023) **DATA DATE 2023**	0.877	(The World Tourism Organization 2022) **DATA DATE 2022**
Trinidad and Tobago	MQ	800,000	(Montero et al. 2023) **DATA DATE 2021**	94,168	(SWMCOL 2023) **DATA DATE 2009**	240	(Solid Waste HUB and Circular Economy 2021) **DATA DATE 2021**	1.4	(World Bank Group 2023) **DATA DATE 2023**	0.217	(The World Tourism Organization 2022) **DATA DATE 2022**
Barbados	HQ	400,000	(Montero et al. 2023) **DATA DATE 2021**	15,000	(Common Seas 2024) **DATA DATE 2021**	17,120	(Solid Waste HUB and Circular Economy 2021) **DATA DATE 2021**	0.29	(World Bank Group 2023) **DATA DATE 2023**	0.079	(The World Tourism Organization 2022) **DATA DATE 2021**
Jamaica	PQ	1,230,000	(Montero et al. 2023) **DATA DATE 2021**	206,640	(Andrade 2024) **DATA DATE 2016**			0.29	(World Bank Group 2023) **DATA DATE 2023**	0.308	(The World Tourism Organization 2022) **DATA DATE 2022**
Belize	PQ	130,000	(Montero et al. 2023) **DATA DATE 2021**	27,500	(OECD 2026) **Data Date 2026**			0.50	(World Bank Group 2023) **DATA DATE 2023**	0.051	(The World Tourism Organization 2022) **DATA DATE 2021**
Haiti	PQ	2,800,000	(Montero et al. 2023) **DATA DATE 2021**	341,000	(OECD 2026) **Data Date 2026**			12.4	(World Bank Group 2023) **DATA DATE 2023**	0.148	(The World Tourism Organization 2022) **DATA DATE 2021**
Brazil	PQ	73,310,000	(Montero et al. 2023) **DATA DATE 2021**			2,162,645	(Solid Waste HUB and Circular Economy 2021) DATA DATE **2021**	215.0	(World Bank Group 2023) **DATA DATE 2023**	3.630	(The World Tourism Organization 2022) **DATA DATE 2022**
Guadeloupe	HQ	346,720	(ORDEC Guadeloupe 2023) **DATA DATE 2023**	7,287	(Banguillot et al. 2023) DATA DATE **2020**	86,680	(ORDEC Guadeloupe 2023) **DATA DATE 2023**	0.379	(National Institue of Statistics and Economic Studies 1990) **DATA DATE 2024**	0.101	(The World Tourism Organization 2022) **DATA DATE 2015**
Puerto Rico	MQ	2,818,165	(Serrano Aquino et al. 2022)	281,817	(EPA 2021) **DATA DATE 2003**	253,635	(Cruz 2021)	3.206	(World Bank Group 2023)	0.182	(The World Tourism

			**DATA DATE 2003**				**DATA DATE 2019**		**DATA DATE 2023**		Organization 2022) **DATA DATE 2022**
Cuba	I/N	946,080	(Pérez 2024) **DATA DATE 2018**	[redacted]	[redacted]	[redacted]	[redacted]	3.20	(World Bank Group 2023) **DATA DATE 2023**	1.614	(The World Tourism Organization 2022) **DATA DATE 2022**
Martinique	PQ	284,000	(L'Institut d'émission des départements d'outre-mer (IEDOM) 2024) **DATA DATE 2023**	996	(Banguillot et al. 2023) **DATA DATE 2020**	[redacted]	[redacted]	0.35	(National Institue of Statistics and Economic Studies 1990) **DATA DATE 2024**	0.003	(The World Tourism Organization 2022) **DATA DATE 2022**

Methodology for Figure 3

Data Collection

The methodology for data collection required for this paper

1. The data variables required were defined and standardized in terms of units of measure or possible categories. For this study, the term "recycled" is defined as any waste that could be repurposed or reused, including (energy, composting, etc.)

Variables for data waste map:

- Total Waste *(Tons per year)*
- Plastic Waste *(Tons per year)*
- Total Waste Recycled *(Tons per year)*
- Plastic Recycled *(Tons per year)*
- Plastic Produced *(Tons per year)*
- Plastic Consumption *(Tons per year)*
- Population *(Total number of people from last census)*
- Tourists *(Total number of people per year)*

2. A shared worksheet was designed to organize the data.

3. The data collection methodology was explained and shared with other authors of the CePlaSt network around the Caribbean and Latin America, who were responsible for finding the information for their country of residence from academic or reliable sources.

Countries included in the scope:

- Dominican Republic
- Colombia
- Panama
- Costa Rica
- Trinidad and Tobago

- Barbados
- Jamaica
- Belize
- Haiti
- Brazil
- Guadeloupe
- Puerto Rico
- Cuba
- Martinique

4. The information collected from other authors was reviewed and organized; if units of measure were different, the necessary conversions were made to match the defined units (tons per day, tons per person, quantities of products produced). If the data were originally published in languages other than English (Spanish or French), we translated them and filled data gaps (e.g., tourism, population) using databases from international or nonprofit organizations.

Table 2*: Simplified data table for Figure 3*

**Country**	*Total Waste (tons per year)*	*Plastic waste (tons per year)*	*Total Recycled (tons per year)*	*Population*	*Tourist (per year)*	*Population + Tourism (POP+TUR)*
Dominican Republic	4,290,000	26,938	[redacted]	11,200,000	7,163,394	18,363,394
Colombia	14,220,000	[redacted]	1,872,774[3]	52,800,000	4,077,639	56,877,639
Panama	1,971,000	204,984	98,550[3]	4,500,000	549,082	5,049,082
Costa Rica	1,620,000	202,500[2]	63,990[3]	5,300,000	876,904	6,176,904
Trinidad and Tobago	800,000	94,168	240[3]	1,400,000	216,893	1,616,893
Barbados	400,000	15,000	17,120[3]	290,000	78,704	368,704
Jamaica	1,230,000	206,640[2]	[redacted]	2,900,000	302,794	3,202,794
Belize	130,000	27,500	[redacted]	500,000	50,546	550,546
Haiti	2,800,000	341,000	[redacted]	12,400,000	148,355	12,548,355
Brazil	73,310,000	[redacted]	2,162,645[3]	215,000,000	3,630,031	218,630,031
Guadelupe	346,720	7,287	86,680	378,561	100,868	479,429
Puerto Rico	2,818,165[1]	281,817[2]	253,635[3]	3,205,680	182,229	3,387,909
Cuba	946,080	[redacted]	[redacted]	3,200,000	1,614,087	4,814,087
Martinique	284,000	996	[redacted]	349,925	3,376	353,301

*The data highlighted in blue were converted to tons per year, given that the sources provide them as percentages of total waste, or units per capita or per day.

[1] Provided in pounds per person per day, converted pounds to tons and multiplied by 365 days (Original value 5.18)

[2] Provided in percentages of total waste (Original values in the respective order: 12.5%, 16.8%, 10%)

[3] Provided in percentages of total waste (Original values in the respective order: 13.17%, 5%, 3.95%, 0.03%, 4.28%, 2.95%, 9%)

Bibliography

Andrade, Danielle. 2024. *A Review of the Solid Waste Management Policy and Recommendations.* Jamaica Environmental Trust. chrome-extension://efaidnbmnnnibpcajpcglclefindmkaj/https://jamentrust.org/download/a-review-of-the-solid-waste-management-policy-and-recommendations/?wpdmdl=20982&refresh=67d4597b74d401741969787.

Banguillot, J, N Chaumond, S Pasquir, and R Touchee. 2023. *Coûts de Gestion Des Déchets d'emballages Ménagers et Papiers Graphiques En Outre-Mer*. https://librairie.ademe.fr/economie-circulaire-et-dechets/6667-couts-de-gestion-des-dechets-d-emballages-menagers-et-papiers-graphiques-en-outre-mer.html.

Centro de Capacitación e Investigación del Plástico. 2022. *Contribución Del Sector Plásticos a La Economía Dominicana*. Elaborado por Despradel y Asociados - DASA. chrome-extension://efaidnbmnnnibpcajpcglclefindmkaj/https://ccip.intec.edu.do/wp-content/uploads/simple-file-list/DASA-Feb-21-23.pdf.

Colombian Ministry of Commerce. 2024. *Informes de Turismo | MINCIT - Ministerio de Comercio, Industria y Turismo*. https://www.mincit.gov.co/estudios-economicos/estadisticas-e-informes/informes-de-turismo.

Common Seas. 2024. "New Barbados National Action Plan, Co-Developed with Common Seas,…." July 16. https://commonseas.com/news/new-barbados-national-action-plan.

Cruz. 2021. "Trash Crisis Leaves Puerto Rico Near 'the Brink.'" Puerto Rico. *Global Press Journal*, February 16. https://globalpressjournal.com/americas/puerto-rico/trash-crisis-leaves-puerto-rico-brink/.

EPA. 2021. *Municipalities Mitigating for Future Disasters Today*. EPA. https://www.epa.gov/system/files/documents/2021-09/gfx-solid-waste-management-in-puerto-rico.pdf.

L'Institut d'émission des départements d'outre-mer (IEDOM). 2024. *INSTITUT D'ÉMISSION DES DÉPARTEMENTS D'OUTRE-MER: Rapport Annuel ÉCONOMIQUE 2023 Martinique*. chrome-extension://efaidnbmnnnibpcajpcglclefindmkaj/https://www.iedom.fr/IMG/pdf/rapport_annuel_2023.pdf.

Montero, Pablo Andrés Alarcón, Salvador Acosta Acevedo, Magda Correal, et al. 2023. "Regional Material Flow Assessment: Municipal Solid Waste EVAL for Latin America and the Caribbean 2023." *IDB Publications*, ahead of print, October 26. Latin America and the Caribbean. https://doi.org/10.18235/0004841.

National Institute of Statistics and Economic Studies. 1990. "DS_Estimation_Population." Population Estimates. https://catalogue-donnees.insee.fr/en/explorateur/DS_ESTIMATION_POPULATION.

Netherlands Enterprise Agency: Ministry of Foreign Affairs. 2021. *Waste Management in the LATAM Region*. Holland Circular Hotspot. https://data.rvo.nl/sites/default/files/2021/02/Report_LATAM_Waste_Management_feb_2021.pdf.

ORDEC Guadeloupe. 2023. *Synthèse des déchets et de l'économie circulaire*. ORDEC. https://www.synergile.fr/wp-content/uploads/2023/11/ORDEC_Dechets_Chiffres_A5_231102.pdf.

Pérez, Tatiana Alonso. 2024. "LA GESTIÓN INTEGRAL DE LOS RESIDUOS SÓLIDOS URBANOS Y LA ECONOMÍA CIRCULAR, UN DESAFÍO PARA EL DESARROLLO LOCAL EN CIENFUEGOS." *Revista cubana de ciencias económicas* 10 (1): 1.

Serrano Aquino, F.V., I.M. Blaggi Vila, and L.E. Rivera Rodriguez. 2022. *Analysis and Recommendations for the Integrated Management of Resources and Waste.* https://estuario.org/wp-content/uploads/2023/04/Analisis-y-recomendaciones-manejo-de-residuos-Estuario.pdf.

Solid Waste HUB and Circular Economy. 2021. “Status of Material Flow Management: Municipal Solid Waste (MSW) for Latin American and the Caribbean.” https://hubresiduoscirculares.org/en/gestion-del-flujo-de-materiales/.
SWMCOL. 2023. *Consulting Services for a Waste Characterization Study in Trinidad and Tobago*. chrome-extension://efaidnbmnnnibpcajpcglclefindmkaj/https://swmcol.co.tt/Portals/0/Documents/News/2023_07_12_PublicPresentation_Final.pdf?ver=4grYXwD2c4QaLD0ICK_2jw%3d%3d.
The World Tourism Organization. 2022. “UN Tourism Data Dashboard, Global and Regional Tourism Performance.” https://www.unwto.org/tourism-data/un-tourism-tracker.
United Nations Development Programme (UNDP). 2023. *Un País Pequeño y Un Reto Enorme: La Gestión Integral de Residuos Sólidos En Panamá*. Panama. https://www.undp.org/es/panama/publicaciones/un-pais-pequeno-y-un-reto-enorme-la-gestion-integral-de-residuos-solidos-en-panama.
World Bank Group. 2023. “Population Total.” https://data.worldbank.org/indicator/SP.POP.TOTL.
